# Shelf-invading low-oxygen waters control Cenozoic organic carbon burial rates

**DOI:** 10.1073/pnas.2526409123

**Published:** 2026-06-22

**Authors:** Rosalind E. M. Rickaby, Thomas J. Wood, Zunli Lu, Christian J. Bjerrum

**Affiliations:** ^a^ Department of Earth Sciences, University of Oxford, Oxford OX1 3AN, United Kingdom; ^b^ Department of Earth and Environmental Sciences, Syracuse University, Syracuse, NY 13244; ^c^ Department of Geosciences and Natural Resource Management, University of Copenhagen, Copenhagen K DK-1350, Denmark

**Keywords:** carbon cycle, sea level, oxygen minimum zone, phosphate, carbon isotopes

## Abstract

The thermostatic mechanisms of Earth’s persistent habitability remain unresolved. High-resolution Cenozoic C isotope records, P accumulation, and coarse-fraction I/Ca allow recalculation and assessment of controls on the global proportion of total carbon buried as organic carbon (*f*_org_), a regulator of atmospheric CO_2_ and O_2_. *f*_org_ was suppressed during the Eocene hothouse, coincident with an oxygenated water column and low water-column phosphate. With decreased sea level, the area for efficient organic carbon and phosphate sedimentary burial diminished, leading increasingly to greater water-column phosphate, higher primary productivity, and emergent water column deoxygenation. The sea-level influence on the areal extent of high sedimentation in shelf regions acts as a control on phosphate availability for new production, respiratory demand, and ocean oxygenation, as proposed by hypsographic models [C. J. Bjerrum, J. Bendtsen, J. J. F. Legarth, *Geochem. Geophys. Geosys.* **7**, 1–24 (2006)]. During intermediate sea-level highs of the Neogene, pulses of enhanced organic carbon burial prevailed for multimillion years, in response to the redox recycling of phosphate when oxygen minimum zones with O_2_ < 90 µmol/kg were present. We propose the existence of a self-limiting intermediate sea-level sweet spot with peak C_org_ burial due to redox recycling of phosphate, whereby oxygen minimum zones (OMZ) with O_2_ < 90 µmol/kg impinge on the most C_org_ rich continental shelf sediments. Such a sweet spot has narrowed over Earth history due to deepening OMZs, stabilizing both atmospheric O_2_ and CO_2_. Continental marine inundation controls on phosphate availability, and the sedimentary carbon flux, provide a positive-feedback and rectifier to perturbations during inception of the icehouse world.

## Significance

Unresolved mechanisms stabilize our planet’s atmosphere. Cenozoic reconstructions of burial rates of organic carbon and phosphorus, and water column oxygenation reveal the first-order feedback between the degree of continental shelf flooding and ocean productivity. Limited aqueous P availability at high sea level, due to efficient continental shelf burial, starved the ocean sedimentary carbon sink, oxygenated the ocean, and led to atmospheric CO_2_ accumulation. Lower sea levels harbored increasing P and emergent deoxygenated waters. The extent of interaction between ocean low-oxygen zones and shelf sediments defines a sea-level “sweet-spot” for rapid burial of organic carbon which acts as a rectifier of glacial conditions. The geological deepening of oxygen minimum zones (OMZs) increasingly stabilized CO_2_ and O_2_ in Earth’s atmosphere.

## Atmospheric Composition Thermostats.

Interacting redox feedbacks have rarely been considered as key drivers of atmosphere and ocean chemistry over the Cenozoic. A temperature-dependent silicate weathering feedback (1) is thought to keep the sources of CO_2_ from the outgassing of volcanoes, and from organic matter weathering, in balance with the sedimentary sinks of CaCO_3_ and organic carbon. Similarly, a thermostat may regulate atmospheric O_2_ since any increase in oxidative weathering drives an increase in the burial rate of C_org_, catalyzed by the redox recycling of P, which self-rectifies due to oxygenation over multimillion year timescales (2). The burial rate of organic carbon acts as a link between these two thermostats and their control on atmospheric CO_2_ and O_2_ by acting as a sink of carbon, and a source of oxygen to the atmosphere on long timescales (3), as well as acting as a fast feedback capable of driving instability in the climate system (4).

On long timescales, the amount of organic carbon produced in the ocean is assumed to be limited by the availability of aqueous phosphorus (P_aq_), supplied by continental weathering (5). Phosphorus is an irreplaceable nutrient utilized by all organisms (6). The intimate linkage between C and P during burial limits extended periods of C_org_ burial in the past due to the efficient removal of P from the water column (7). P_aq_ is buried in three main forms: organic-, iron-, and calcium-associated P, collectively referred to as P_reac_. Under anoxic bottom waters, the burial of organic and/or iron associated P is suppressed, resulting in C_org_/P_reac_ increasing from ~50 in bioturbated muds under oxic conditions to ~250 in laminated muds under anoxic conditions (8–12) Also, bulk sedimentation rate and the C_org_/P_reac_ have a nonlinear relationship with the highest C_org_:P_reac_ ratios at intermediate sedimentation rates (13–15). Under anoxic conditions, the reduction of iron oxy-hydroxides to ferrous iron in solution releases phosphate (10). Consequently, phosphate recycling to the water column is greater from carbon-rich sediments overlain by oxygen-depleted waters at intermediate sedimentation rates on the outer shelf and upper slope (8, 10, 13, 16, 17).

**Fig. 1.**
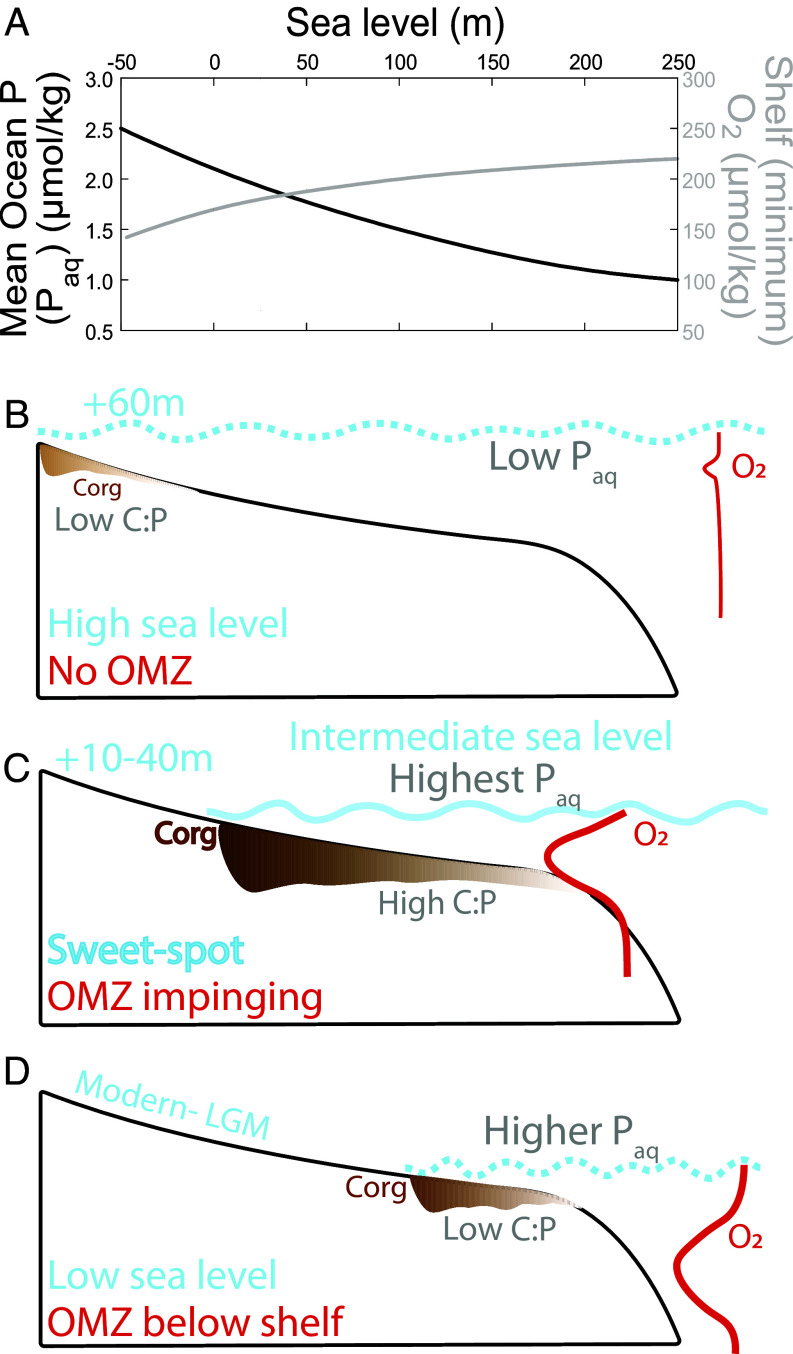
The relationship between sea level, P_aq_ (black), and water column minimum O_2_ (gray) as found by hypsographic modeling plotted as solid curves (*A*), redrawn from steady state model results of Bjerrum et al. (figure 6C from ref. 7). Panels (*B*–*D*): C_org_ burial rates are indicated by the brown shading with annotations to indicate the C_org_/P_reac_ of that burial, with low C_org_/P_reac_ at the high and low sedimentation rates of the coastal and pelagic realms, but high C_org_/P_reac_ at intermediate sedimentation rates (12, 14, 15). P_reac_ is most efficiently buried at the highest sea levels (>+50 m) with the greatest area of shallow coastal sediments, yielding the lowest P_aq_, which restricts organic carbon production globally (*A*). At sea-levels <+50 m, P_aq_ is more available for the production of C_org_, which increases the respiratory burden of the water column, leading to the emergence of an oxygen minimum zone. At water column O_2_ concentrations <90 µmol/kg, P_aq_ release from sediments is triggered which allows extensive C_org_ burial. This is most effective when those minimum O_2_ waters still impinge on the C_org_-rich sediments of the continental shelf at intermediate sea levels of +10 to +40 m (*C*). At lower sea levels still (<+10 m), the OMZs drop beneath the continental shelf and lie in contact with much lower C_org_ content sediments, restricting P_reac_ release, as is the case in the modern ocean (*D*). Further, the flux of P_aq_ from weathering may be limited due to the cold conditions in the glaciated world.

The importance of the degree of inundation of the shelf as an amplifying-feedback on climate has been considered in terms of the enhanced alkalinity of a lower sea-level ocean coined the “coral reef hypothesis” (18, 19) and the impact of decreased sea level on carbon and phosphate burial on shelves during glacials (20). At low sea level, more nutrients have been proposed to have been transferred to the deep ocean, resulting in higher P_aq_ and less dissolved oxygen (Fig. 1). Bjerrum et al. (7) used hypsographic modeling to show that a sea level decrease of order of 200 m is sufficient to change the residence time of phosphate from ~10 ky to ~50 ky and to more than double the average P_aq_ concentration due to the decreased area available for efficient C_org_ and P_org_ burial in the shallowest sediments (Fig. 1 *A*, *B*, and *D*). Such a sea-level dependence of P_aq_ availability on C_org_ and P_org_ burial is increasingly invoked to account for a significant fraction of the CO_2_ oscillation during glacial-interglacial cycles (21–23).

Here, we demonstrate that the hypsographic models of seawater compositional change due to sea level, and the intimate coupling between the oxygen, phosphorus, and carbon cycles is supported by data through the Cenozoic Era. The sedimentary burial rate of carbon as both carbonate and organic carbon was significantly reduced during the Eocene hothouse (56 to 33.9 Ma), coincident with the period of highest atmospheric CO_2_ and suggestive that an imbalance between weathering source and sedimentary sink led to an accumulation of carbon in the atmosphere. At this time, the ocean was highly oxygenated, as evidenced from I/Ca. Coincidently, low P in pelagic deep-sea sediments indicated an extended period of phosphate starvation which inhibited primary production, reduced the sedimentary sink of carbon, and plausibly drove an imbalance in the carbon cycle of the Eocene hothouse world.

## Results

### Reconstruction of Fractional Burial Rates of Organic Carbon.

The size of the sedimentary organic carbon reservoir over time plays a fundamental role in the long-term oxidation of the Earth’s surface environment (24). Due to the ~25‰ C isotopic fractionation associated with fixation of carbon via Rubisco (25, 26), the carbon isotopic composition of the ocean provides a uniquely global measure of the proportional rates of C_org_ burial relative to the CaCO_3_ sink, assuming the isotopic composition of the carbon input remains quasi-constant (27). The isotopic composition of the carbon input may change (*SI Appendix*, *SI Text*) due to kerogen weathering (28) or to variance in the solid Earth degassing signature (29). The near consistency of the average ocean C isotopic composition at +2‰ throughout a billion years of geological history suggests that the input has remained largely constant at ~−6‰ (30) despite significant perturbations in tectonism, exposure of older sediments and the terrestrial biota.

**Fig. 2.**
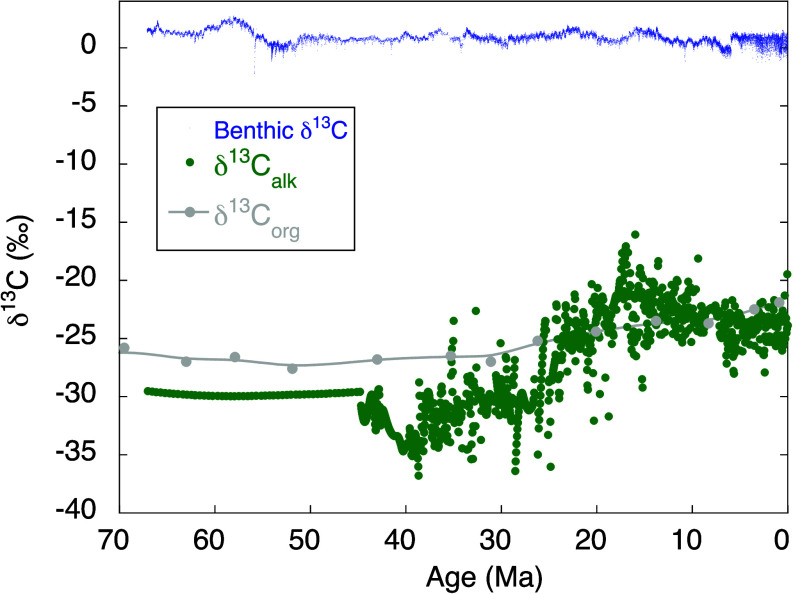
Compiled records of carbon isotopes: benthic foraminifera, blue, (34); alkenones, green, corrected for the 4.2 ‰ offset from biomass (33, *SI Appendix*, *SI Text*) including interpolated low-resolution phytane (35) for the oldest parts of this record. A longer-term compilation of δ^13^C of C_org_, gray, from ref. 27 is plotted as interpolated data to obtain a common timescale.

Mass balances of C fluxes are often calculated assuming that organic matter is buried with a δ^13^C ~ −25‰ lighter than the ocean. This fractionation factor is measured in the Rubisco enzyme isolated from spinach and is not representative of algal Rubisco fractionation, which may have evolved to be as small as ~ -11‰ (31). To circumvent uncertainties in the magnitude of the fractionation associated with the diversity of affinity of Rubisco for CO_2_ fixation, it is possible to compile a record of the δ^13^C of marine C_org_ which implicitly accounts for a variable fractionation factor, rather than relying on the assumption of a fractionation factor. A new compilation, used for the reconstruction of *p*CO_2_ (32), also provides the most complete and continuous dataset of alkenone δ^13^C (Fig. 2), a molecule exclusively produced by distinct species of coccolithophore. Alkenone δ^13^C has been shown to be consistently offset by 4.2‰ from the isotopic composition of the bulk cellular biomass (33; *SI Appendix*, *SI Text*) and provides a measure of the δ^13^C of marine sedimentary C_org_ without contamination from terrestrial sources.

Since approximately 30 Ma, the carbon isotope fractionation between alkenone δ^13^C (δ^13^C_alk_) and oceanic DIC has decreased from ~24 to ~16‰, from extrapolation of pre- and post-30 Ma data to a zero intercept (*SI Appendix*, Fig. S1). Such a trend toward a heavier isotopic composition of marine C_org_ toward the modern is also captured at lower resolution by Hayes et al. (27, Fig. 2). Insight into size-dependent intracellular reservoirs of carbon (36) suggests that the alkenone-producing coccolithophores have adapted on geological timescales to diminishing carbon availability by diminishing in size (37, 38), with a trade-off to faster division rate. This size adaptation also shrinks the internal cellular carbon pool but keeps it full, which allows a relaxation in the affinity of their Rubisco enzyme (39) with a concomitant reduction in enzymatic C isotopic fractionation (26, 40). As a result, the alkenone record, which is not species-specific, likely reflects the assemblage shift toward smaller, faster-growing species with a relaxed Rubisco affinity for carbon and a diminished Rubisco fractionation factor. That the change in fractionation factor is ~10‰ could reflect a switch in carbon substrate used from CO_2_ to HCO_3_^−^.

The fractional burial flux of organic carbon *C_org_* relative to total carbon (*f*_*org*_) added to marine sediments for a given interval is

$$f_{\mathrm{org}}=\left[\frac{\delta_{\text{in}}-\delta_{\text{carb}}}{\delta_{\text{org}}-\delta_{\text{carb}}}\right],$$

where δ_carb_ is δ^13^C of carbonate deposition, δ_org_ is the δ^13^C of *C*_org_ deposition, and δ_in_ is the mean isotopic composition of inputs to the ocean. Although Kump and Arthur (41) suggest the use of the shallow record of carbonate δ^13^C for mass balance calculation, these records tend to suffer from bias and vital effects (42, 43). The high-resolution benthic δ^13^C splice of Westerhold et al. (34) is used as a measure of the δ^13^C_carb_ with a bulk carbonate to benthic offset of 0.9‰ (28).

The records of δ^13^C_org_ and δ^13^C_carb_ were interpolated at ~50 ky intervals to provide datasets at comparable resolution and age before calculation of *f*_org_. Linear regression between the fractionation and δ^13^C_carb_ (27) suggests that δ_in_ is poorly constrained by the data but approximates −6‰ in accord with Hayes et al. (27, *SI Appendix*, *SI Text*), and is close to the canonical value of volcanic degassing inputs (29). We recognize that δ_in_ variation may have some significance through the Cenozoic, but find its variation through time unconstrained with the current unknowns. For simplicity, we treat δ_in_ as a constant (*SI Appendix*, Fig. S2).

**Fig. 3.**
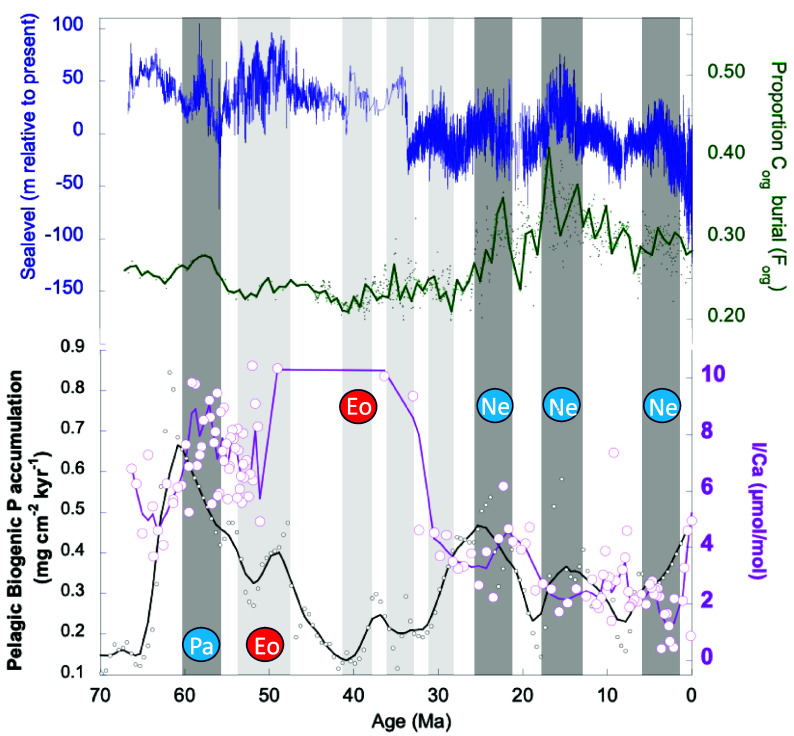
Records of sea level (55), blue; reconstructed proportional burial flux of C_org_ based on the alkenone δ^13^C record showing small green individual datapoints and an interpolated curve; an interpolated record of I/Ca of the coarse-fraction from site 1264 spliced with 1262 in the South Atlantic (open purple circles) (this study) and pelagic biogenic P accumulation in sediments with <5 wt% Al_2_O_3_, a proxy for P_aq_ (black, 56). The dark gray bars highlight the periods of time with higher proportional C_org_ burial, higher sea levels, higher pelagic biogenic P accumulation, and lower water column oxygen concentration. The light gray bars highlight the periods of higher sea-level fluctuations unmatched by C_org_ burial. During this period, pelagic biogenic P accumulation is low and water-column I/Ca is high. The red and blue circles denote the events (Paleocene Pa, Eocene Eo, and Neogene, Ne), which are then plotted in Fig. 5. The age model is from Westerhold et al. (34).

The *f*_org_ record shows a number of peaks and troughs with a multimillion-year wavelength varying in amplitude around a mean value of about a third of the carbon sink buried as organic carbon (Fig. 3). The main peaks in *f*_org_ are associated with sea-level highs, but not all sea-level highs are associated with peaks of relative organic carbon burial. Most notably, the *f*_org_ peak amplitude relative to sea-level prior to 23 Ma is more muted than during the sea-level highs in the later Oligo-Miocene (23 to 5.3 Ma). The individual peaks in *f*_org_ are supported by additional sedimentary evidence. The peak centered around 6 Ma coincides with the “biogenic bloom” (44–46), and that around 15 Ma with the Monterey event associated with large-scale economic hydrocarbon reservoirs (47, 48). The Paleocene–Eocene is known as a time of enhanced C_org_ burial (49, 50) and high inferred productivity. High C_org_ burial characterized the Oligo-Miocene as documented to harbor 12.5% of the world’s oil reservoirs (~23 Ma) (51, 52). The rise in *p*O_2_ associated with pulses of *f*_org_, is coincident with, and could have permitted, significant nonallometric shifts in brain-body size of mammals and birds in the aftermath of the K/Pg extinction during the Paleocene and early Neogene (~23 Ma) (53, 54).

The Eocene suppression in *f*_org_ from the average of 0.3 down to ~0.2, for an extended period from ~50 to 30 Ma, is also supported by the identification of “missing C_org_” in Eocene marine sediments, where C_org_ burial was depressed by an order of magnitude compared to modern (57). Overall, our record suggests that an increase in *f*_org_ is generally associated with sea-level rise during the Cenozoic, except for the extended period from ~50 to ~30 Ma when the proportional burial rates are suppressed significantly. This period is characterized, in part, by some of the highest sea levels of the last ~65 My at over 60 m above current sea levels during the Early Eocene.

Our reconstructed changes in *f*_org_, calculated using a methodology sensitive to global burial rates, challenge earlier estimates based on sedimentary budgets (23, 58). In todays’ oceans, the burial rate of C_org_ is highly laterally heterogeneous with up to 70% of burial constrained to narrow but areally extensive, high-sedimentation continental shelf regions (7, 14). Sedimentary budgets are susceptible to regional bias and suffer from challenges in the accuracy of accumulation rates that are necessarily derived from sediment thickness between two age points, each of which contains uncertainty.

### Sea-Level Mediated Phosphate and Organic Carbon Burial.

A first-order driver of the *f*_org_ record is the sea-level dependence on the availability of P_aq_ (7, 59). The lowest pelagic biogenic P accumulation rates [from Follmi (56)], a proxy for global P_aq_, occurred between 45 Ma and 32 Ma, approximately coinciding with the period of lowest *f*_org_ (Fig. 3). Higher sea levels resulted in a reduced global ocean P_aq_ due to efficient C_org_ and P_reac_ burial on the associated wider continental shelf. In contrast, lower sea levels resulted in elevated P_aq_ due to burial with a higher C_org_/P_reac_ on the minimal continental shelf and upper slope. P_aq_-limitation of global productivity associated with high sea level therefore may account for the first-order observation of depressed proportional C_org_ productivity and burial during the highest sea levels of the Eocene (56 to 33.9 Ma) relative to the rest of the Cenozoic, a scenario that persisted for many millions of years.

That low pelagic biogenic P accumulation approximates P_aq_ is well supported by an association between low pelagic biogenic P accumulation and other indicators of oligotrophy. The increasing oligotrophy of the calcifying taxa through the Eocene (60) and a predominance of large heavily calcified forms [e.g., Claxton et al. (43)], characterized by low growth rates, are suggestive of adaptation to low nutrients and high CO_2_ conditions. The pelagic biogenic P accumulation rates also suggest an extended period of P_aq_ limitation during the late Cretaceous (100 to 66 Ma). The Late Cretaceous was also dominated by phytoplankton adapted to oligotrophic conditions when sea level was thought to still be ~100 m above present (61, 62).

**Fig. 4.**
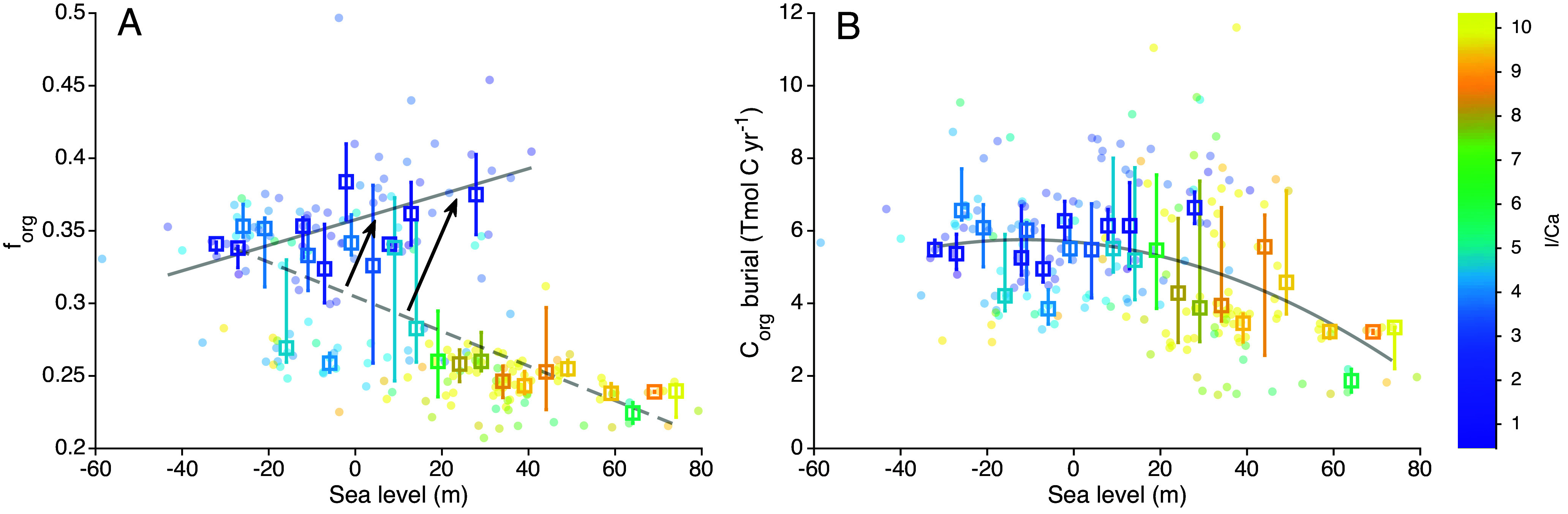
Organic carbon cycle response to changes in Eustatic sea level. (*A*) Scatter of proportional C_org_ burial (*f*_org_) against sea level (55), colored by the I/Ca ratio. *f*_org_ data were split above and below the I/Ca = 2.3 OMZ threshold of ref. 64, binned at 5 m sea-level intervals, and plotted as box (median) and whiskers (IQR) for bins with n > 2 datapoints, which were colored by I/Ca. Linear trendlines were drawn for *f*_org_ data at low I/Ca ≤ 2.3 (solid, r = 0.557, *P* = 2.5e-03) and at high I/Ca > 2.3 (dashed, r = −0.543, *P* = 2.7e-14). Black arrows show the approximate direction of sea-level associated perturbations from 23 Ma on in Fig. 3. (*B*) Scatter of calculated C_org_ burial flux against sea level, colored by I/Ca, a proxy for ocean oxygen levels, and overlain with boxes and whiskers as in (*A*). C_org_ burial flux data were overlain with a best-fit second-order polynomial, plotted between min and max sea level boxes. Note how organic burial tends toward an optimum between 0 and +40 m.

Collectively, there are indications of higher *f*_org_ at intermediate sea levels (+0 to +40 m) (Fig. 4). The maintenance of a higher-than-average *f*_org_ for an extended multimillion-year timescale is challenging due to the short residence time of P_aq_ in the ocean (15 to 100 ky) (16). In order to explain the apparently extended pulses of *f*_org_ over 3 to 5 My accompanying sea-level highs outside of the Eocene high-stand, it is necessary to appeal to mechanisms that allowed replenishment of phosphate into the water column. This P sustained prolonged multimillion-year periods of C_org_ burial, via a redox sensitive recycling of phosphorus and elevated C_org_/P_reac_ burial ratio (7–9, 16, 17, 63), which also led to more organic carbon burial per unit P delivered to the ocean.

### Oxygen Minimum Zones (OMZ) Presence, P Availability, and Sea Level.

It is possible to test this mechanism of redox-sensitive recycling of P as a driver of extended C_org_ burial using planktic foraminiferal I/Ca, a proxy for past water column O_2_ concentrations (61). The principle of the proxy is that the speciation of iodine between the I^-^ ion and the IO_3_^−^ ion is sensitive to O_2_ availability, such that the reduction of IO_3_^−^ to I^−^ is often observed in OMZs. It is only the IO_3_^−^ ion that substitutes for the CO_3_^2−^ and is incorporated into calcium carbonate fossils such as planktic foraminifera. Higher I/Ca ratios, in principle, indicate higher degrees of water column oxygenation. Existing core-top data show that planktic I/Ca < 2.3 µmol/mol in water columns represents O_2_ beneath <90 µmol/kg (65–67); similar to the oxygen concentration threshold, related to anoxia in shallow porewaters, proposed for the release of P_reac_ back to the water column (7). Therefore, planktic I/Ca can serve as an independent constraint on the connection between P_reac_ accumulation rates and the reconstructed fractional C_org_ burial (Fig. 3).

We have measured the coarse-fraction (mixed species planktic foraminifera) I/Ca over the Cenozoic from Southern Atlantic Ocean (ODP Site 1262 and 1264). I/Ca is highest during the period of 30 to 50 Ma with values of ~8 to 10 µmol/mol, and trends to lower values from 30 Ma toward the modern day, with additional fluctuations above the ~2 µmol/mol background to values of ~4 µmol/mol at ~20 Ma, 10 Ma, and in the last 2 to 3 My. The high I/Ca in the Eocene (56 to 33.9 Ma), suggest that the O_2_ content of the upper water column from 30 to 48 Ma could have exceeded 140 µmol/kg, based on a core-top study (64), qualitatively indicating that OMZs were absent from these sites during the Eocene (Fig. 3). The decline in I/Ca to reach 2.3 µmol/mol sporadically suggests that OMZs with water column O_2_ < 90 µmol/kg started to emerge and intensify at lower sea levels from the Oligocene onward.

Taking I/Ca as a measure of the oxygenation of the water column, during the Eocene, the water column oxygen content was elevated above the critical threshold of 90 µmol/kg. At such high water-column O_2_ concentrations, phosphate is adsorbed onto iron oxyhydroxides which, along with C_org_ burial, results in a low C_org_/P_reac_ burial ratio with phosphate efficiently buried in coastal sediments. Due to the highest sea levels from 33 to 48 Ma, at about +50 to +66 m above present-day sea level, C_org_ production, and water column O_2_ consumption was limited by P_aq_ (7, Fig. 1). Consequently, the ocean harbored the lowest productivity and the water column became highly oxygenated in accord with the N isotopic signature during Cenozoic warm periods (68).

Since 30 Ma, water column deoxygenation emerged in response to declining sea levels below ~+40 m, as indicated by a decrease in I/Ca, and was driven toward a threshold of oceanic oxygen concentration < 90 µmol/kg. (Figs. 3 and 4*A*). These observations agree with model results in which lower sea levels resulted in reduction of the shelf area with high sedimentation rates for efficient C_org_ and P_reac_ burial. This resulted in an increase in P_aq_, potentially amplified by an increase in P weathering input (69, 70) resulting in greater productivity of C_org_ and a greater respiratory burden for the water column (7) (Fig. 1*A*).

Increasing P_aq_ though the Neogene is further supported by the fossil record. Experiments indicate that coccolithophores grown at high P_aq_ decrease their calcification rate (71) because cell division becomes too fast for the diffusive carbon supply rate for calcification, such that calcite/cell decreases (72). Since the Eocene (56 to 33.9 Ma), coccolithophores have shrunk (37), with higher growth rates (73) and lower calcification intensities (43), suggestive of a community change in response to increasing P_aq_ and declining *p*CO_2_. The fossil record of Cenozoic diatom size echoes this trend, so all plankton may respond in a similar way to elevated P_aq_ (74).

**Fig. 5.**
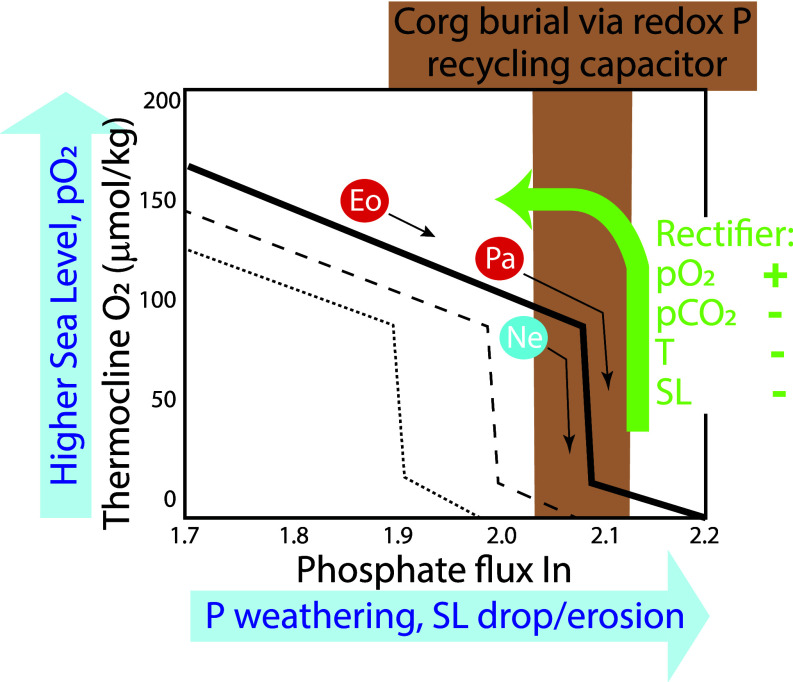
Schematic adapted from the transient model simulations of (*SI Appendix*, figure S12*B* from ref. 7) to show the minimum water-column oxygen concentration vs. the input phosphate whereby thermocline waters close to a critical oxygen concentration (~90 µmol/kg), with a small additional input of P (black arrows), can lead to a threshold behavior in C_org_ burial. This critical oxygen concentration is sensitive to the weathering inputs of phosphate, which dictate the oxygen demand of organic matter produced; or to the temperature dependence of the solubility of oxygen in the water column, or to the atmospheric oxygen content. As such the relationship between the O_2_ threshold and sea level is not absolute but varies, dependent on these drivers of the background seawater chemistry. Small P increases to the ocean from erosion or residence time changes associated with sea-level rise or fall can be amplified through the redox release of P from coastal sediments and lead to a significant increase in organic carbon burial. Also plotted in the red and blue circles is the approximate position of the climate system before the sea-level rise events identified in Fig. 3 with respect to the threshold, with arrows indicating the perturbation by a small P input. For short-term perturbations, the system naturally rectifies by burying sufficient carbon to either decrease sea level and move the OMZs off the continental shelf, or decreasing temperature which raises the O_2_ content of the water column. On longer timescales, the build-up of O_2_ from the burial of C_org_, raises the O_2_ content of the water column and rectifies the system. Also shown, in gray, are hypothetical curves of how the threshold may move with lower atmospheric O_2_ content, or much warmer water columns with lower O_2_ concentrations due to solubility. Although this schematic suggests that the ocean tends to anoxia in the OMZs with even greater P input, as discussed and shown in Fig. 1, it is likely that the deepening of the OMZs beyond the continental shelf, as revealed by the data here, represents a lower threshold to the behavior.

During the Neogene, increasing P_aq_ drove areas of the ocean close to but above the threshold O_2_ content of ~90 µmol/kg (Figs. 4*A* and 5). Superimposed on the background trend, were perturbations. Each individual period of sea-level rise of up to +40 m above modern, was associated with an extended multimillion-year pulse of *f*_org_, lower I/Ca ratios, and higher P availability (Figs. 3 and 4*A*). An incremental P input from an increase in weathering or from sea-level rise-associated coastal erosion was sufficient to push the ocean beneath the critical oxygen threshold (90 µmol/kg). Crossing this threshold triggered disproportionate phosphate release from sedimentary organic- and iron-associated P_reac_ (Fig. 1*C*) yielding carbon burial with a high C_org_/P_reac_ and extended periods of high C_org_ burial (Fig. 4).

During the final stages of the Neogene, as sea level on average decreased below ~+10 m, *f*_org_ decreased, and the amplitude of perturbations diminished while I/Ca remained low (Figs. 3 and 4*A*). These observations suggest that once beneath the O_2_ threshold (I/Ca ~ 2.3) for effective P regeneration, P_aq_ is high and small perturbations to the P inventory can no longer be amplified.

### An Organic Carbon Burial Sweet Spot.

Our Cenozoic data indicate that the relationship between eustatic sea level and *f*_org_ is nonlinear, with two trends separated by a threshold I/Ca of ~2.3 µmol/mol (64), with an instability and hysteresis in *f*_org_ at intermediate sea-levels (+0 to −40 m). Beneath this O_2_ threshold, with I/Ca < 2.3 µmol/kg, *f*_org_ decreases with decreasing sea level (Fig. 4*A*). The two trends in *f*_org_ as a function of sea level, converge at a sea level of ~0 to −20 m, and tend toward lower *f*_org_ with further sea level decrease, associated with lower oxygen values, in agreement with high-resolution Pleistocene data (Fig. 4*A* and *SI Appendix*, Fig. S3).

The nonlinear trend of *f*_org_ as a function of sea level is paralleled by the calculated burial flux of organic carbon, where a sweet spot optimum occurs at intermediate sea levels (Fig. 4*B*, see *SI Appendix*). One interpretation of this sweet spot optimum is that the low sea level side of the optimum is associated with a slow-down in weathering supply of P_aq_ during cooling and ice-sheet growth under colder climatic conditions. Such a weathering reduction would have caused a decrease in P_aq_, diverting the runaway P_aq_-rich and anoxic ocean predicted by Bjerrum’s model with a nondynamic P influx from continents (7) (Figs. 1*A* and 5). By contrast, the observed continuous benthic foraminifera record suggests that the deep ocean remained oxic during the Cenozoic.

Alternatively, P_aq_ may have been reduced at low sea levels due to vertical migration of the OMZs relative to P_reac_ rich shelf sediments. For phosphate to be remobilized by OMZs and catalyze extended periods of ocean productivity, then the OMZs must impinge on P_reac_ rich sediments, predominantly on the continental shelf, yielding the highest rate of redox-dependent release of phosphate from those organic-rich sediments (Fig. 1). With greater sea-level recession, the OMZs may drop deeper than the depths of the P_reac_-rich sediments of the continental shelf. This is the situation in the modern ocean, where the OMZs are deeper than the continental shelf (75) and associated with low sedimentation rates (11), limiting the potential P regeneration. In our record, the optimum for the OMZ to coincide with C_org_-rich continental shelf sediments and propagate C_org_ burial appears to be close to ~+10 to +40 m above modern sea level (Fig. 2). At such intermediate sea levels, the top of the OMZ [at present at ~−100 to −150 m (76)], would impinge fully on the modern continental shelf (−120 m to 0 m at present), allowing a maximum of C_org_ burial. In addition, colder ocean conditions associated with low sea level would further have deepened the OMZs through the temperature dependence of remineralization rates (77, 78).

### Carbon Cycle Feedbacks.

Sea-level control on P_aq_ acts together with a sedimentation rate and redox-sensitive C_org_/P_reac_ to change the gearing of C_org_ burial per mole of P supplied from weathering to create a sweet spot of high C burial rates at intermediate sea levels (16, 79 and Fig. 4*B*). The total burial of organic carbon can change with sea level even though the input and burial of P_reac_ may remain unchanged (7).

Near the O_2_ threshold, the redox-dependent acceleration of C_org_ burial acts as a fast positive-feedback (4, 80), hastening the onset of glaciation, or as a dynamic rectifier. Any perturbation to higher sea levels leads to extensive C_org_ burial, which drives the climate back to a cooler state. For example, the inundated continental shelf of interglacial periods yields higher C_org_ burial rates, more positive δ^13^C (81), and a natural oscillation back to a lower CO_2_ glaciation (20, 82, 83). Over the Cenozoic, the maximum change in the proportional C_org_ flux, from 0.21 to 0.35, represents only an approximate 20% increase in the *total* C sink. The C burial events persist for <5 My (Fig. 3) and are small enough and insufficiently long-lived to drive an imbalance with weathering supply and toward a runaway icehouse (84). But the C burial events may be sufficiently dynamic to maintain a resilient icehouse against smaller warming perturbations, demanding a driver to a sea level higher than +40 m, to return to persistent greenhouse conditions (Fig. 4*A*).

The intermediate shelf-area sweet spot for high organic C burial rates is inherently self-limiting due to the drawdown in atmospheric CO_2_. Associated cooling and ice-sheet growth deepens the OMZs through decreased temperature or sea level, sliding the OMZs off the continental shelf and deeper than the optimal P_reac_ release, thus reducing C_org_ burial (Fig. 1*C*). Alternatively, redox-driven P_reac_ release may be limited by a slower negative-feedback due to elevated atmospheric oxygen from high organic carbon burial that reoxygenates the ocean (2, 9) invoked to generate self-sustaining oscillations (79). This oscillation may apply to the Cenozoic cycles in proportional organic C burial, with an approximate wavelength of ~5 My, after the Eocene period of P_aq_ starvation.

### Implications for the Total Sedimentary Carbon Sink.

**Fig. 6.**
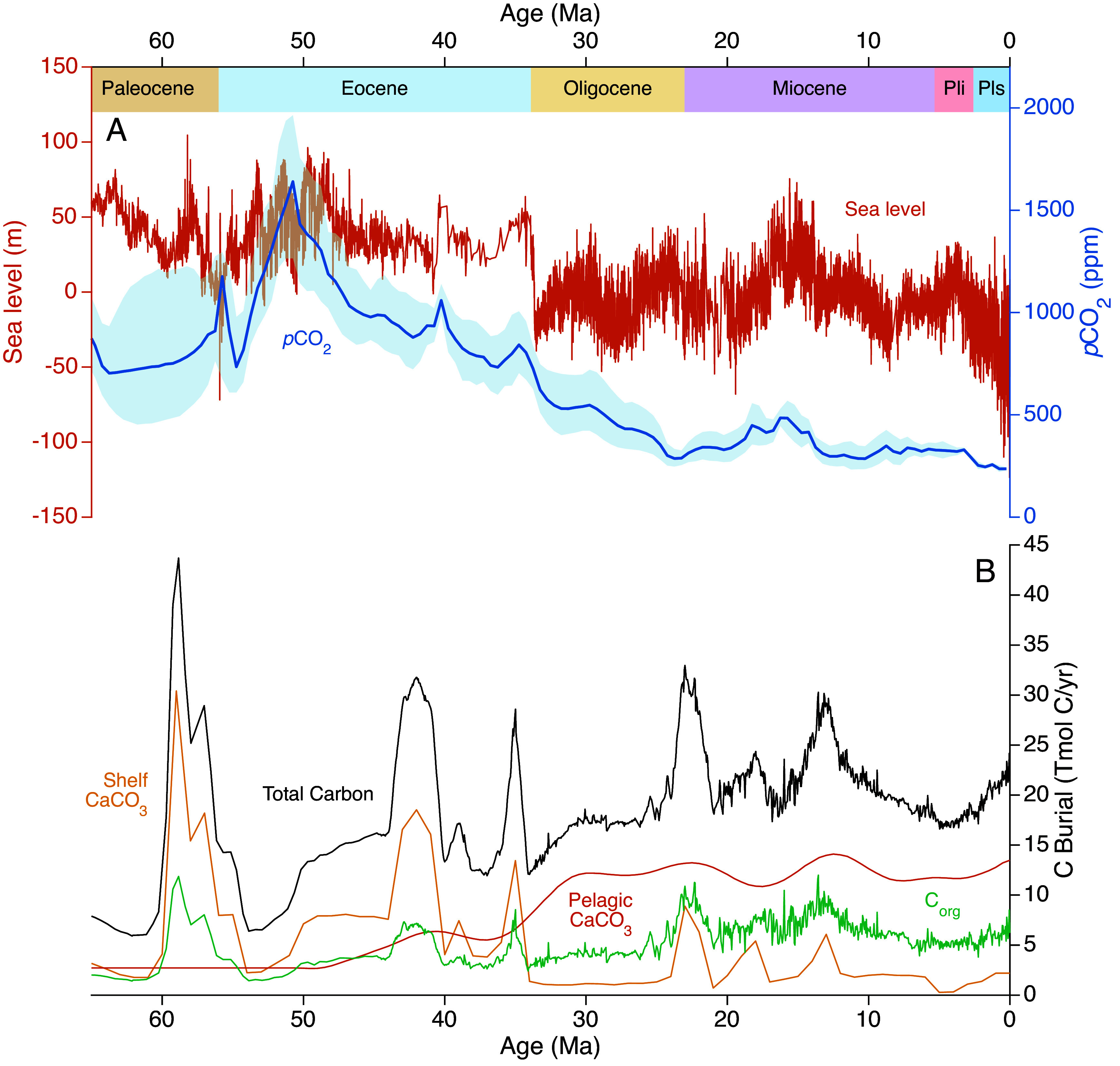
(*A*) Cenozoic-sea level from Miller et al. (55) and atmospheric *p*CO_2_ from CenCO2PIP (32). (*B*) Total Cenozoic C burial (black, *SI Appendix*, *SI Text*), reconstructed from the sum of shelf carbonate burial (yellow, 87) and pelagic carbonate burial (orange, 90) divided by (1 - *f*_org_). C_org_ burial (green) was reconstructed from total C burial multiplied by *f*_org_.

Given the link between P_aq_ limitation of the C_org_ flux to sediments and high CO_2_ in the atmosphere during the Eocene (56 to 33.9 Ma), a deceleration in the sedimentary carbon sink may have contributed to an accumulation of ocean-atmosphere CO_2_. A more detailed C_org_ flux calculation, accounting for shelf (85–87) and pelagic CaCO_3_ accumulation (88, 89), suggests that, to a first order, the Eocene experienced limited carbon burial (*SI Appendix*, *SI Text* and Fig. 6). This reduced carbon sink was terminated by an uptick in shelf C flux starting at ~43 Ma, coincident with a rise in the ^87^Sr/^86^Sr isotope ratio (69, 70). P_aq_ starvation of the ocean was increasingly alleviated from ~35 Ma onward, promoting a higher total sedimentary flux of both C_org_ and CaCO_3_ (Fig. 6 and *SI Appendix*, *SI Text*). Pelagic carbonate appears responsive to excess carbon and alkalinity from shelf loss (88, 89) and P_aq_ availability, such that the decline in tropical shelf accumulation (85–87) through the Paleogene is compensated approximately by a similar magnitude increase in the pelagic C flux through the Neogene but with additional sedimentary C pulses when water column P was higher (Fig. 6). Sea level represents a carbon-cycle-feedback via its influence on the redox-dependent availability of P_aq_, to increase the carbon sink and act as a positive-feedback toward ice-sheet inception, but reduces carbon sinks when ice sheets are larger.

### Geological Implications.

The sweet spot that allows enhanced organic carbon burial for multimillions of years through redox recycling of P requires a rather unique set of geological ingredients: sufficient P weathering, poising the ocean near the O_2_ threshold at 90 µmol/kg in the water column, and a depth of the O_2_ minimum that impinges on plentiful organic-rich sediments on an extensive continental shelf (Fig. 5). Significant low-latitude continental land mass, tectonism (91), or life on land to accelerate P_aq_ input (92), and a biological pump dominated by eukaryotes (93, 94), yielding an OMZ, may be prerequisites for significant atmospheric O_2_ build up, inevitably accompanied by significant glaciation. For any given sea level and phosphate supply, in a past lower *p*O_2_, shallow OMZs would have impinged at shallower depths of the high sedimentation-rate continental shelf, typically containing a higher fraction of C_org_ and P_reac_ for redox recycling. Consequently, the ocean would have been more closely poised to the threshold to allow extensive C_org_ burial for small changes in sea level or perturbations to the P budget (95, 96). With shallower OMZs, there was a much greater areal extent of the continental shelves for persistent phosphate release during sea-level retreat, allowing greater redox recycling of P for longer, before inhibition of the organic carbon burial cycle by build-up of atmospheric oxygen. Such a mechanism speaks to the pulsed nature of O_2_ build-up and would have resulted in much higher amplitude glaciations, possibly contributing to the potential for Snowball Earth (97). Glaciations have become more muted as O_2_ has accumulated in the atmosphere and since the deepening of the OMZs across the Mesozoic boundary (98) due to the rise of the mineralized plankton. The sweet spot for organic carbon burial has narrowed over time, increasingly stabilizing the climate and atmosphere (96).

## Conclusions

Records of *f*_org_ are consistent with a persistent positive-feedback on the size of the carbon sink where ocean oxygen levels decrease as the Earth became more glaciated during the Cenozoic. The Eocene-ocean (56 to 33.9 Ma) was well oxidized and starved of P_aq_ by the degree of continental shelf inundation, effectively trapping the P with highly efficient shallow C_org_ burial. With progressive glaciation and declining sea levels, the reduced area of continental shelf inundation resulted in P_aq_ increasing in availability, elevating the oxygen demand of the water column until the OMZs emerged in the aftermath of the Eocene/Oligocene boundary (~30 Ma). The presence of subsurface waters with an O_2_ content less than 90 µmol/kg that impinge on the C_org_ and P_reac_ rich sediments of the continental shelf represents a rapid-feedback on carbon burial. At sea-level perturbations ~+10 to +40 m above modern, C_org_ burial persists for timescales >1 My due to the redox recycling of phosphate; a scenario which is likely self-limiting. The presented records demonstrate that pulsed burial of organic carbon is likely a persistent feature and rectifier of glaciated worlds. At lower sea levels still, less than +10 m, the sediment release of P is more limited when the OMZs are deeper than the continental shelf break. As a result, the modern ocean reflects a more anoxic water column than that experienced in the Eocene and likely through the Mesozoic, which counterintuitively harbored the most oxygenated waters, coincident with the warmest periods and highest sea levels. The deepening of the OMZs through time, has been a key factor in stabilizing the climate of the Earth system.

## Materials and Methods

### I/Ca Analysis.

ODP Sites are located on the Walvis Ridge with ODP Site 1262 from 27° 11.15’ S 1°34.62’ E, 4,755 m water depth, and ODP Site 1264 from 28° 31.95’S 2° 50.73’ E and 2,505 m water depth. 3 to 5 mg of coarse-fraction material (> 63 μm) for each sample was weighed, crushed, and rinsed with deionized water to remove residual porewater before dissolution. A previous study (99) showed that the coarse-fraction, without further cleaning by oxidative or reductive reagents, was sufficient to capture the same trend in I/Ca as cleaned, single-genus planktic foraminiferal measurements. I/Ca was measured on a quadrupole inductively coupled plasma mass spectrometer (Bruker M90) at Syracuse University. Carbonate samples were dissolved in 3% nitric acid and diluted to form solutions with ~50 ppm Ca for analyses. Fresh calibration standards, matching the sample matrix, were prepared for every batch of samples. The precision of ^127^I is typically better than 1% and is not reported separately for each sample. The long-term accuracy is guaranteed by frequent repeats of the reference material JCp-1. The detection limit of I/Ca is typically below 0.1 μmol/mol.

## Supplementary Material

Appendix 01 (PDF)

Dataset S01 (XLSX)

## Data Availability

Study data are included in the article and/or supporting information.

## References

[1] J. C. G. Walker, P. B. Hays, J. F. Kasting, A negative feedback mechanism for the long-term stabilization of Earth’s surface temperature. J. Geophys. Res. Oceans **86**, 9776–9782 (1981), 10.1029/JC086iC10p09776.

[2] P. Van Capellan, E. D. Ingall, Redox stabilization of the atmosphere and oceans by phosphorus-limited marine productivity. Science **271**, 493–496 (1996). 11541251 10.1126/science.271.5248.493

[3] Z. Lu, R. E. M. Rickaby, J. L. Payne, A. N. Prow, Phanerozoic co-evolution of O2-CO2 and ocean habitability. Natl. Sci. Rev. **11**, nwae099 (2024). 38915915 10.1093/nsr/nwae099 PMC11194836

[4] D. Hülse, A. Ridgwell, Instability in the geological regulation of Earth’s climate. Science **389**, eadh7730 (2025), 10.1126/science.adh7730. 40997180

[5] R. E. Hecky, P. Kilham, Nutrient limitation of phytoplankton in freshwater environments: A review of recent evidence on the effects of enrichment. Limnol. Oceanogr. **33**, 796–822 (1988).

[6] C. R. Benitez-Nelson, The biogeochemical cycling of phosphorus in marine systems. Earth Sci. Rev. **51**, 109–135 (2000).

[7] C. J. Bjerrum, J. Bendtsen, J. J. F. Legarth, Modelling organic carbon burial during sea-level rises with application to the Cretaceous. Geochem. Geophys. Geosys. **7**, 1–24 (2006).

[8] E. Ingall, R. Jahnke, Evidence for enhanced phosphorus regeneration from marine sediments overlain by oxygen depleted waters. Geochim. Cosmochim. Acta **58**, 2571–2575 (1994).

[9] P. Van Cappellen, E. D. Ingall, Benthic phosphorus regeneration, net primary production, and ocean anoxia: A model of the coupled marine biogeochemical cycles of carbon and phosphorus. Paleoceanogr. Paleoclimatol. **9**, 677–692 (1996).

[10] A. S. Colman, H. D. Holland, “The global diagenetic flux of phosphorus from marine sediments to the oceans: Redox sensitivity and the control of atmospheric oxygen levels” in Marine Authigenesis: From Global to Microbial, C. R. Glenn, L. Prévôt-Lucas, J. Lucas, Eds. (SEPM Special Publication, 2000), **vol. 66**, pp. 53–75.

[11] D. E. Canfield, C. J. Bjerrum, S. Zhang, H. Wang, X. Wang, The modern phosphorus cycle informs interpretations of Mesoproterozoic Era phosphorus dynamics. Earth Sci. Rev. **208**, 103267 (2020).

[12] T. J. Algeo, E. Ingall, Sedimentary Corg: P ratios, paleocean ventilation, and Phanerozoic atmospheric pO2. Palaeogeogr. Palaeoclimatol. Palaeoecol. **256**, 130–155 (2007).

[13] E. D. Ingall, R. M. Bustin, P. Van Cappellen, Influence of water column anoxia on the burial and preservation of carbon and phosphorus in marine shales. Geochim. Cosmochim. Acta **57**, 303–316 (1993).

[14] E. D. Ingall, P. van Capellan, Relation between sedimentation rate and burial of organic phosphorus and organic carbon in marine sediments. Geochem. Cosmochim. Acta **54**, 373–386 (1990).

[15] L. D. Anderson, M. L. Delaney, K. L. Faul, Carbon to phosphorus ratios in sediments: Implications for nutrient cycling. Global Biogeochem. Cycles **15**, 65–79 (2001), 10.1029/2000GB001270.

[16] K. C. Ruttenberg, “The global phosphorus cycle” in Treatise on Geochemistry, H. D. Holland, K. K. Turekian, Eds. (Elsevier, New York, 2003), **vol. 8**, pp. 585–643.

[17] K. Wallmann, Feedbacks between oceanic redox states and marine productivity: A model perspective focused on benthic phosphorus cycling. Glob. Biogeochem. Cycles **17**, 1084 (2003), 10.1029/2002GB001968.

[18] W. H. Berger, Increase of carbon dioxide in the atmosphere during deglaciation: The coral reef hypothesis. Naturwissenschaften **69**, 87–88 (1982).

[19] B. N. Opdyke, J. C. G. Walker, Return of the coral reef hypothesis: Basin to shelf partitioning of CaCO_3_ and its effect on atmospheric CO_2_. Geology **20**, 733–736 (1992). 11538164 10.1130/0091-7613(1992)020<0733:rotcrh>2.3.co;2

[20] W. S. Broecker, Glacial to interglacial changes in ocean chemistry. Prog. Oceanogr. **11**, 151–197 (1982).

[21] K. Wallmann, B. Schneider, M. Sarnthein, Effects of eustatic sea-level change, ocean dynamics, and nutrient utilization on atmospheric pCO2 and seawater composition over the last 130 000 years: A model study. Clim. Past **12**, 339–375 (2016), 10.5194/cp-12-339-2016.

[22] O. Cartapanis, D. Bianchi, S. L. Jaccard, E. D. Galbraith, Global pulses of organic carbon burial in deep-sea sediments during glacial maxima. Nat. Commun. **7**, 10796 (2016), 10.1038/ncomms10796. 26923945 PMC4773493

[23] O. Cartapanis, E. D. Galbraith, D. Bianchi, S. L. Jaccard, Carbon burial in deep-sea sediment and implications for oceanic inventories of carbon and alkalinity over the last glacial cycle. Clim. Past **14**, 1819–1850 (2018).

[24] J. M. Hayes, J. R. Waldbauer, The carbon cycle and associated redox processes through time. Philos. Trans. R. Soc. B Biol. Sci. **361**, 931–950 (2006). 10.1098/rstb.2006.1840 PMC1578725 16754608

[25] K. H. Freeman, J. M. Hayes, Fractionation of carbon isotopes by phytoplankton and estimates of ancient CO2 levels. Glob. Biogeochem. Cycles **6**, 185–198 (1992), 10.1029/92GB00190. 11537848

[26] S. Poudel , Biophysical analysis of the evolution of substrate specificity in RuBisCO. Proc. Natl. Acad. Sci. U.S.A. **48**, 30451–30457 (2020). 10.1073/pnas.2018939117 PMC7720200 33199597

[27] J. M. Hayes, H. Strauss, A. J. Kaufman, The abundance of ^13^C in marine organic matter and isotopic fractionation in the global biogeochemical cycle of carbon during the past 800 Ma. Chem. Geol. **161**, 103–125 (1999).

[28] L. A. Derry, Closing the geologic carbon cycle. Proc. Natl. Acad. Sci. U.S.A. **121**, e2409333121 (2024). 39374393 10.1073/pnas.2409333121 PMC11494303

[29] E. Mason, M. Edmonds, S. Turchyn, Remobilization of crustal carbon may dominate volcanic arc emissions. Science **357**, 290–294 (2017), 10.1126/science.aan5049. 28729507

[30] D. H. Rothman, J. M. Hayes, R. E. Summons, Dynamics of the Neoproterozoic carbon cycle. Proc. Natl. Acad. Sci. U.S.A. **100**, 8124–8129 (2004). 10.1073/pnas.0832439100 PMC166193 12824461

[31] A. J. Boller, P. J. Thomas, C. M. Cavanaugh, K. M. Scott, Low stable carbon isotope fractionation by coccolithophore RubisCO. Geochim. Cosmochim. Acta **75**, 7200–7207 (2011).

[32] B. Hönisch ; Cenozoic CO2 Proxy Integration Project (CenCO2PIP) Consortium, Toward a Cenozoic history of atmospheric CO_2_. Science **382**, eadi5177 (2023), 10.1126/science.adi5177. 38060645

[33] E. B. Wilkes, R. B. Y. Lee, H. L. O. McClelland, R. E. M. Rickaby, A. Pearson, Carbon isotope ratios of coccolith–associated polysaccharides of Emiliania huxleyi as a function of growth rate and CO_2_ concentration. Org. Geochem. **119**, 1–10 (2018).

[34] T. Westerhold , An astronomically dated record of Earth’s climate and its predictability over the last 66 million years. Science **369**, 1383–1387 (2020). 32913105 10.1126/science.aba6853

[35] C. R. Witkowski , Molecular fossils from phytoplankton reveal secular P_co2_ trend over the Phanerozoic. Sci. Adv. **4**, eaat4556 (2018), 10.1126/sciadv.aat4556. 30498776 PMC6261654

[36] N. Chauhan, R. E. M. Rickaby, Size-dependent dynamics of the internal carbon pool drive isotopic vital effects in calcifying phytoplankton. Geochim. Cosmochim. Acta **373**, 35–51 (2024).

[37] S. Herrmann, H. R. Thierstein, Cenozoic coccolith size changes–Evolutionary and/or ecological controls? Palaeogeogr. Palaeoclimatol. Palaeoecol. **333–334**, 92–106 (2012), 10.1016/j.palaeo.2012.03.011.

[38] B. Suchéras-Marx, J. Henderiks, Downsizing the pelagic carbonate factory: Impacts of calcareous nannoplankton evolution on carbonate burial over the past 17 million years. Glob. Planet. Change **123**, 97–109 (2014), 10.1016/j.gloplacha.2014.10.015.

[39] J. N. Young, R. E. M. Rickaby, M. Kapralov, D. Filatov, Adaptive signals in algal Rubisco reveal a history of ancient atmospheric CO_2_. Phil. Trans. Roy. Soc. **367**, 483–492 (2012). 10.1098/rstb.2011.0145 PMC3248704 22232761

[40] G. G. B. Tcherkez, G. D. Farquhar, T. J. Andrews, Despite slow catalysis and confused substrate specificity, all ribulouse bisphosphate carboxylases may be nearly perfectly optimised. Proc. Natl. Acad. Sci. U.S.A. **103**, 7246–7251 (2006). 16641091 10.1073/pnas.0600605103 PMC1464328

[41] L. R. Kump, M. A. Arthur, “Global chemical erosion during the Cenozoic: Weatherability balances the budget” in Tectonic Uplift and Climate Change, W. Ruddiman, Ed. (Plenum, New York, 1997), pp. 399–426, 10.1007/978-1-4615-5935-1.

[42] C. Bolton, H. Stoll, Late Miocene threshold response of marine algae to carbon dioxide limitation. Nature **500**, 558–562 (2013), 10.1038/nature12448. 23985873

[43] L. M. Claxton, H. L. O. McClelland, M. Hermoso, R. E. M. Rickaby, Eocene emergence of highly calcifying coccolithophores despite declining atmospheric CO_2_. Nat. Geosci. **15**, 826–831 (2022).

[44] L. Diester-Haass, K. Billups, K. C. Emeis, In search of the late Miocene–early Pliocene “biogenic bloom” in the Atlantic Ocean (Ocean Drilling Program Sites 982, 925, and 1088). Paleoceanography **20**, 20 (2005), 10.1029/2005PA001139.

[45] M. E. Gastaldello , The Late Miocene-Early Pliocene biogenic bloom: An integrated study in the Tasman Sea. Paleoceanogr. Paleoclimatol. **38**, e2022PA004565 (2023), 10.1029/2022pa004565.

[46] B.T. Karatsolis, B. C. Lougheed, D. D. Vleeschouwer, J. Henderiks, Abrupt conclusion of the late Miocene-early Pliocene biogenic bloom at 4.6-4.4 Ma. Nat. Commun. **13**, 353 (2022), 10.1038/s41467-021-27784-6. 35039500 PMC8764042

[47] E. S. C. Anttila , Timing and tempo of organic carbon burial in the Monterey Formation of the Santa Barbara Basin and relationships with Miocene climate. Earth Planet. Sci. Lett. **620**, 118343 (2023), 10.1016/j.epsl.2023.118343.

[48] L. A. Derry, C. France-Lanord, Neogene growth of the sedimentary organic carbon reservoir. Paleoceanography **11**, 267–275 (1996), 10.1029/95PA03839.

[49] K. Kurtz, L. R. Kump, M. A. Arthur, J. C. Zachos, A. Paytan, Early Cenozoic decoupling of the global carbon and sulfur cycles. Paleoceanography **18**, 1–14 (2003), 10.1029/2003pa000908.

[50] N. Komar, R. E. Zeebe, G. R. Dickens, Understanding long-term carbon cycle trends: Late Paleocene through the early Eocene. Paleoceanogr. Paleoclimatol. **28**, 650–662 (2013), 10.1002/palo.20060.

[51] L. Diester-Haass, K. Billups, K. Emeis, Enhanced paleoproductivity across the Oligocene/Miocene Boundary as evidenced by benthic foraminiferal accumulation rates. Paleogeogr. Paleoclimatol. Paleoecol. **302**, 464–473 (2011).

[52] H. D. Klemme, G. F. Ulmishek, Effective petroleum source rocks of the world: Stratigraphic distribution and controlling depositional factors. Bull. Am. Assoc. Petrol. Geol. **75**, 1809–1851 (1991).

[53] J. B. Smaers , The evolution of mammalian brain size. Sci. Adv. **7**, eabe2101 (2021), 10.1126/sciadv.abe2101. 33910907 PMC8081360

[54] R. E. M. Rickaby, T. J. Wood, R. Gollnisch, C. Bjerrum, Z. Lu, Indicators and evolutionary consequences of the Cenozoic history of Oxygen. Cold Spring Harb. Perspect. Biol. (2026), 10.1101/cshperspect.a041923 (accepted).

[55] K. G. Miller , Cenozoic sea-level and cryospheric evolution from deep-sea geochemical and continental margin records. Sci. Adv. **6**, eaaz1346 (2020), 10.1126/sciadv.aaz1346. 32440543 PMC7228749

[56] K. B. Follmi, The phosphorus cycle, phosphogenesis and marine phosphate-rich deposits. Earth Sci. Rev. **40**, 55–124 (1996).

[57] A. Olivarez Lyle, M. W. Lyle, Missing organic carbon in Eocene marine sediments: Is metabolism the biological feedback that maintains end-member climates. Paleoceanogr. Paleoclim. **21**, PA2007 (2006). 10.1029/2005PA001230.

[58] Z. Li, Y. G. Zhang, M. Torres, B. J. W. Mills, Neogene burial of organic carbon in the global ocean. Nature **613**, 90–95 (2023), 10.1038/s41586-022-05413-6. 36600067

[59] C. J. Bjerrum, J. Bendtsen, Relations between long term sea-level change, shelf-ocean exchange and shelf burial of organic material. Eos Trans. AGU **83**, Ocean Sci. Meet.Suppl., Abstract OS41G-08 (2002).

[60] J. D. Asanbe, J. Henderiks, Major shifts in Equatorial Atlantic and Pacific calcareous Nannofossil assemblages across the Early Eocene Climatic Optimum (EECO; 53–49 Ma). Paleoceanogr. Paleoclimatol. **40**, e2024PA005038 (2025), 10.1029/2024PA005038.

[61] R. M. Leckie, T. J. Bralower, R. Cashman, Oceanic Anoxic events and plankton evolution: Biotic response to tectonic forcing during the mid-Cretaceous. Paleoceanogr. Paleoclim. **17**, e2001PA000623 (2002), 10.1029/2001PA000623.

[62] S. Tozzi, O. Schofield, P. Falkowski, Historical climate change and ocean turbulence as selective agents for two key phytoplankton functional groups. Mar. Ecol. Prog. Ser. **274**, 123–132 (2004).

[63] C. P. Slomp, J. Thompson, G. De Lange, Enhanced regeneration of phosphorus during formation of the most recent Eastern Mediterranean sapropel (S1). Geochim. Cosmochim. Acta **66**, 1171–1184 (2002).

[64] X. Wang, R. E. M. Rickaby, Z. Lu, A deep dive into the planktic foraminiferal I/Ca in global core-tops. Glob. Planet. Change **261**, 105412 (2026).

[65] Z. Lu, H. C. Jenkyns, R. E. M. Rickaby, I/Ca ratios in marine carbonate as a palaeo-redox proxy during oceanic anoxic events. Geology **38**, 1107–1110 (2010).

[66] Z. Lu , Oxygen depletion recorded in upper waters of the glacial Southern Ocean. Nat. Commun. **7**, 11146 (2016), 10.1038/ncomms11146. 27029225 PMC4821880

[67] W. Lu , Refining the planktic foraminiferal I/Ca proxy: Results from the Southeast Atlantic Ocean. Geochim. Cosmochim. Acta **287**, 318–327 (2020).

[68] A. Auderset , Enhanced ocean oxygenation during Cenozoic warm periods. Nature **609**, 77–82 (2022). 36045236 10.1038/s41586-022-05017-0 PMC9433325

[69] J. K. Caves, A. B. Jost, K. V. Lau, K. Maher, Cenozoic carbon cycle imbalances and a variable weathering feedback. Earth Planet. Sci. Lett. **450**, 152–163 (2016), 10.1016/j.epsl.2016.06.035.

[70] J. Veizer , 87Sr/86Sr, δ13C and δ18O evolution of Phanerozoic seawater. Chem. Geol. **161**, 59–88 (1999).

[71] K. Kayano, Y. Shiraiwa, Physiological regulation of coccolith polysaccharide production by phosphate availability in the coccolithophorid Emiliania huxleyi. Plant Cell Physiol. **50**, 1522–1531 (2009), 10.1093/pcp/pcp097. 19587028

[72] S. Gill, Q. Zhang, G. M. Henderson, R. E. M. Rickaby, The physiological response of contrasting coccolithophore species to Ocean Alkalinity Enhancement. J. Geophys. Res. Biogeosci. **130**, e2025JG009103 (2025), 10.1029/2025JG009103.

[73] K. Billups, R. E. M. Rickaby, D. P. Schrag, Cenozoic pelagic Sr/Ca records: Exploring a link to paleoproductivity. Paleoceanography **19**, PA3005 (2004).

[74] Z. V. Finkel, M. E. Katz, J. D. Wright, O. M. Schofield, P. G. Falkowski, Climatically driven macroevolutionary patterns in the size of marine diatoms over the Cenozoic. Proc. Natl. Acad. Sci. U.S.A. **102**, 8927–8932 (2005), 10.1073/pnas.0409907102. 15956194 PMC1157017

[75] J. Karstensen, L. Stramma, M. Visbeck, Oxygen mimumum zones in the eastern tropical Atlantic and Pacific oceans. Progr. Oceanogr. **77**, 331–350 (2008).

[76] A. Paulmier, D. Ruiz-Pino, Oxygen minimum zones (OMZs) in the modern ocean. Prog. Oceanogr. **80**, 113–128 (2009).

[77] C. M. Marsay , Attenuation of sinking particulate organic carbon flux through the mesopelagic ocean. Proc. Natl. Acad. Sci. U.S.A. **112**, 1089–1094 (2015), 10.1073/pnas.1415311112. 25561526 PMC4313834

[78] E. Y. Kwon, F. Primeau, J. L. Sarmiento, The impact of remineralization depth on the air-sea carbon balance. Nat. Geosci. **2**, 630–635 (2009), 10.1038/ngeo612.

[79] I. C. Handoh, T. M. Lenton, Periodic mid-Cretaceous oceanic anoxic events linked by oscillations of the phosphorus and oxygen biogeochemical cycles. Global Biogeochem. Cycles **17**, 1092 (2003), 10.1029/2003GB002039.

[80] C. J. Bjerrum, Sea level, climate, and ocean poisoning by sulfide all implicated in the first animal mass extinction. Geology **46**, 575–576 (2018).

[81] K. I. C. Oliver , A synthesis of marine sediment core δ^13^C data over the last 150 000 years. Clim. Past **6**, 645–673 (2010).

[82] M. Adloff, A. Jeltsch-Thommes, F. Poppelmeier, T. F. Stocker, F. Joos, Sediment fluxes dominate glacial-interglacial changes in ocean carbon inventory: Results from factorial simulations over the past 780000 years. Clim. Past **21**, 571–592 (2025), 10.5194/cp-21-571-2025.

[83] A. C. Fowler, R. E. M. Rickaby, E. Wolff, Exploration of a simple model for ice ages. GEM - Int. J. Geomathematics **4**, 227–297 (2012), 10.1007/s13137-012-0040-7.

[84] Y. Godderis, Y. Donnadieu, B. J. W. Mills, What models tell us about the evolution of carbon sources and sinks over the Phanerozoic. Annu. Rev. Earth Planet. Sci. **51**, 471–492 (2023), 10.1146/annurev-earth-032320-092701.

[85] B. N. Opdyke, B. H. Wilkinson, Surface area control of shallow cratonic to deep marine carbonate accumulation. Paleoceanography **3**, 685–703 (1988), 10.1029/PA003i006p00685.

[86] T. Salles , Carbonate burial regimes, the Meso-Cenozoic climate, and nannoplankton expansion.. Proc. Natl. Acad. Sci. U.S.A. **122**, e2516468122 (2025), 10.1073/pnas.2516468122. 41343679 PMC12704742

[87] J. M. Husson, S. E. Peters, Shifting carbonate burial between oceanic and continental crust across Earth history. Earth Planet. Sci. Lett. **677**, 119810 (2026), 10.1016/j.epsl.2025.119810.

[88] G. J. A. Brummer, A. J. M. Eijden, “Blue-ocean” paleoproductivity from pelagic carbonate mass accumulation rates. Mar. Micropal. **19**, 99–117 (1992).

[89] A. Dutkiewicz, R. D. Müller, The history of Cenozoic carbonate flux in the Atlantic Ocean constrained by multiple regional carbonate compensation depth reconstructions. Geochem. Gephys. Geosys. **23**, e2022GC010667 (2002), 10.1029/2022GC010667.

[90] B. P. Boudreau, Y. Luo, Retrodiction of secular variations in deep-sea CaCO3 burial during the Cenozoic. Earth Planet. Sci. Lett. **474**, 1–12 (2017), 10.1016/j.epsl.2017.06.005.

[91] C. R. Walton , Evolution of the crustal phosphorus reservoir. Sci. Adv. **9**, eade6923 (2023), 10.1126/sciadv.ade6923. 37146138 PMC10162663

[92] B. Peret , Root architecture responses: In search of phosphate. Plant Physiol. **166**, 1713–1723 (2014). 25341534 10.1104/pp.114.244541 PMC4256877

[93] N. J. Butterfield, Oxygen, animals and aquatic bioturbation: An updated account. Geobiology **16**, 3–16 (2018), 10.17863/CAM.21972. 29130581

[94] T. M. Lenton, R. A. Boyle, S. W. Poulton, G. A. Shields-Zhou, N. J. Butterfield, Co-evolution of eukaryotes and ocean oxygenation in the Neoproterozoic Era. Nat. Geosci. **7**, 257–265 (2014).

[95] M. S. Dodd , Marine phosphorus and atmospheric oxygen were coupled during the Great Oxidation Event. Nat. Commun. **16**, 9151 (2025), 10.1038/s41467-025-64194-4. 41093832 PMC12528410

[96] A. Bachan , A model for the decrease in amplitude of carbon isotope excursions across the Phanerozoic. Am. J. Sci. **317**, 641–676 (2017), 10.2475/06.2017.01.

[97] P. F. Hoffman, D. P. Schrag, The Snowball Earth hypothesis: Testing the limits of global change. Terra Nova **14**, 129–155 (2002).

[98] W. Lu , Late inception of a persistently oxygenated upper ocean. Science **361**, eaar5372 (2018). 10.1126/science.aar5372. 29853552

[99] X. Zhou, E. Thomas, R. E. M. Rickaby, A. M. E. Winguth, Z. Lu, I/Ca evidence for upper ocean deoxygenation during the Paleocene-Eocene Thermal Maximum (PETM). Paleoceanography **29**, 964–975 (2014), 10.1002/2014PA002702.

