# Supplementary material for: Shelf-invading low-oxygen waters control Cenozoic organic carbon burial rates
Source: Proc Natl Acad Sci U S A. 2026 Jun 22;123(26):e2526409123. doi: 10.1073/pnas.2526409123 (PMC13320727; doi:10.1073/pnas.2526409123)
Supplement: Supplementary file 1 — Appendix 01 (PDF) [file pnas.2526409123.sapp.pdf]

PNAS

Supporting Information for
Shelf invading low oxygen waters denote sweet-spot for the organic carbon sink

Rosalind E. M. Rickaby[1], Thomas J. Wood[1], Zunli Lu[2] and Christian J. Bjerrum[3]

*Rosalind E. M. Rickaby.
Email: rosr@earth.ox.ac.uk

This PDF file includes:

Supporting text
Figures S1 - S3

Supporting Information Text

Section 1: Calculation of Change in Total Sedimentary C Flux

In order to translate the proportional C_{org} flux into a quantitative flux, it is necessary to constrain the total flux of carbon out of the ocean. This can be done by assuming that the carbon flux out of the ocean matches that of the weathering flux into the ocean and the system is at steady state with no imbalance. The Cenozoic weathering flux has been modelled to decrease (1), increase (2) or stay approximately the same (3) over the Cenozoic and so it is hard to constrain (4). Alternatively, it is possible to compile records of carbonate outputs from pelagic records (5, 6) and integrate with records of shelf carbonate accumulation (7, 8, 9, 10) which broadly concur. We have used the sedimentary neritic $CaCO_3$ burial rates from (10) and the pelagic $CaCO_3$ burial rates from (6). Any missing values from these records for 0-65 Ma were filled with nearest neighbour values. The neritic and pelagic $CaCO_3$ burial records were summed to give total $CaCO_3$ burial, linearly interpolated at the resolution of the f_{org} record, and divided by $(1 – f_{org})$ to obtain total C burial. This could thereafter be multiplied by f_{org} to give the C_{org} burial flux. The C_{org} burial rate then reflects the "fast" response of the carbon cycle which can drive an imbalance between CO_2 source and CO_2 sink.

With the oversimple assumption that the apparent partitioning of carbon between the shelf and the deep ocean results in a constant total $CaCO_3$ accumulation leads to an equation that shows the implication of a change in our proportional burial for the total carbon burial sink (T):

$0.34T_1 + 0.66T_1 = T_1$
$0.21T_2 + 0.79T_2 = T_2$

If the larger proportional burial, the flux of carbonate, remains constant as it is partitioned between the shelf and the deep-sea then:

$$\frac{T_1}{T_2} = \frac{0.79}{0.66} = 1.20$$

Section 2: Exploring the sensitivity of f_{org} to parameters

Marine algal carbon isotopic fractionation is sensitive to the same factors; growth rate, CO_2, size, across different phytoplankton groups (11, 12) so the trends in the alkenone $\delta^{13}C$ records approximate the $\delta^{13}C$ of marine organic carbon buried. The two dominant parameters which may vary and could alter the calculated f_{org} are the isotopic fractionation between alkenone $\delta^{13}C$ ($\delta^{13}C_{alk}$) and cellular $\delta^{13}C$ ($\delta^{13}C_{org}$), and the $\delta^{13}C$ of the input ($\delta^{13}C_{in}$) which integrates both volcanic degassing and weathering of different carbon reservoirs. A third factor, the proportion and isotopic composition of C_{org} burial by the terrestrial biosphere is beyond the scope of this study but is generally considered to be the minor proportion of the total organic burial (10-20%) (13).

For the isotopic fractionation between alkenone $\delta^{13}C$ and biomass $\delta^{13}C$ ($\Delta\delta^{13}C_{org}$- $\delta^{13}C_{alk}$), original estimates were placed at 3.8 ‰ (14). This value was later re-estimated at 4.24 ± 0.05 ‰ and shown to be invariant with growth rate (15). This value has been used ubiquitously in all pCO_2 reconstructions based on $\delta^{13}C_{alk}$ (16) similar to the value reported by the CenCO2PIP consortia (4.68±0.78 ‰) (17). This value has been confirmed by later chemostat experiments and again shown invariant with environmental conditions but a range between 3.5-5.2 ‰ quoted (18). This range is explored for its impact on f_{org} giving a low sensitivity (Δf_{org} of 0.005) for a change in $\Delta\delta^{13}C_{org}$- $\delta^{13}C_{alk}$ of ~ 0.7 ‰. (Fig. S2). We note that all of these determinations of $\Delta\delta^{13}C_{org}$- $\delta^{13}C_{alk}$ were performed on *G. huxleyi* yet the majority of the record (>273 ka) is dominated by its alkenone producing ancestors that may have expressed a different fractionation factor. Given that the biochemical role of the alkenones within the cell and the metabolic network remains the same on

evolutionary timescales, it is likely that the isotopic offsets have not changed significantly (19). The overall agreement between the low resolution record of $\delta^{13}C_{org}$ (20) and our inferred $\delta^{13}C_{org}$ from $\delta^{13}C_{alk}$ suggests that our assumption of a constant offset may be reasonable.

The $\delta^{13}C_{in}$ was found to be -6.1 ‰ across the Cenozoic from type II regression analysis (21, see method below), which is inline with regressions on the binned and interpolated data which suggest an input of -5.5 ‰ for the Paleogene. There is a hint in this regression of a marginally lighter input (-5.8 ‰) during the Neogene which may be consistent with the inference of an impact from kerogen weathering on the carbon input driving the $\delta^{13}C_{in}$ lighter (towards – 8‰ (± 1.9 ‰) in 4). In both (4) and our calculations, light $\delta^{13}C_{in}$ ~ -8 yields very high f_{org} values of up to 0.5, that seems unrealistic. We show the sensitivity of calculated f_{org} to variations in $\delta^{13}C_{in}$ and find a Δf_{org} of 0.04 per 1 ‰ $\Delta\delta^{13}C_{in}$. We have used a constant value of -6 ‰ for consistency with the range of volcanic inputs and which yields a similar Cenozoic average f_{org} of 0.3 (20). Continental weathering recycles both carbonate and organic carbon outputs of previous eras, and subduction of different ratios of carbonate and organic carbon, results in differences in derived volcanic isotope composition. For example, a shift in $\delta^{13}C_{in}$ of – 2‰ around 25 Ma could minimize the elevated f_{org} around that time. However, such a scenario appears unlikely since changes in carbon sources by tectonic uplift/subduction would tend to evolve on longer timescales, and the isotopic composition of weathering matches prior volcanic sources over million-year timescales such that $d^{13}C_{in}$ would tend to be buffered against change. The change in slope of our regression analysis further supports an increase in f_{org} in the Neogene relative to the Paleocene (Fig. S2). Such secular changes are unlikely to account for the shorter-term features of increased f_{org} associated with sea-level highstands. A short-term isotopic lightening of $\delta^{13}C_{in}$ on the order of a few ‰ could account for these features but it is more likely that the $\delta^{13}C_{in}$ became isotopically lighter at sea-level lowstands on exposure and weathering of the isotopically light C_{org} rich shelf sediments.

Method for Binning and Regression Analysis

We use the benthic record of $\delta^{13}C_{carb}$ from (22) corrected with an offset of +0.9±0.2 ‰ following Derry and references therein. $\delta^{13}C_{alk}$ is taken from the CenCO2PIP compilation (17). An isotope offset between alkenone and organic biomass is assumed to 4.68±0.78 ‰ following compilation in CenCO2PIP Table S3. Phytane is assumed to be offset by 3.6±1.44 ‰ as in CenCO2PIP.

We bin the $\delta^{13}C_{carb}$ and $\delta^{13}C_{alk}$ into separate 300 kyr long time bins and calculate the mean values for each bin. The variance of each bin is calculated as the variance of the $\delta^{13}C$ values within the bin (and not as the mean of the variances of the values in the bin). We consider this approach to best represent the true global and temporal variance within the bin interval (Supplementary data).

Empty bins are filled by linear interpolation. If there are less than three values in a bin interval, we assign a variance to the bin that is 2 times the mean variance of the full time series. We split the two binned timeseries at 25 Ma, because of the marked major change in $\delta^{13}C_{alk}$. Regression analyses are then calculated separately for bins before or after 25 Ma. We use a “York” regression (23) taking into account the variance of each point in both x and y direction. Note that the slope and y-intercept of the “Paleogene” is very dependent on the extrapolation of the alkenone record and should be viewed as suggestive only.

Figures

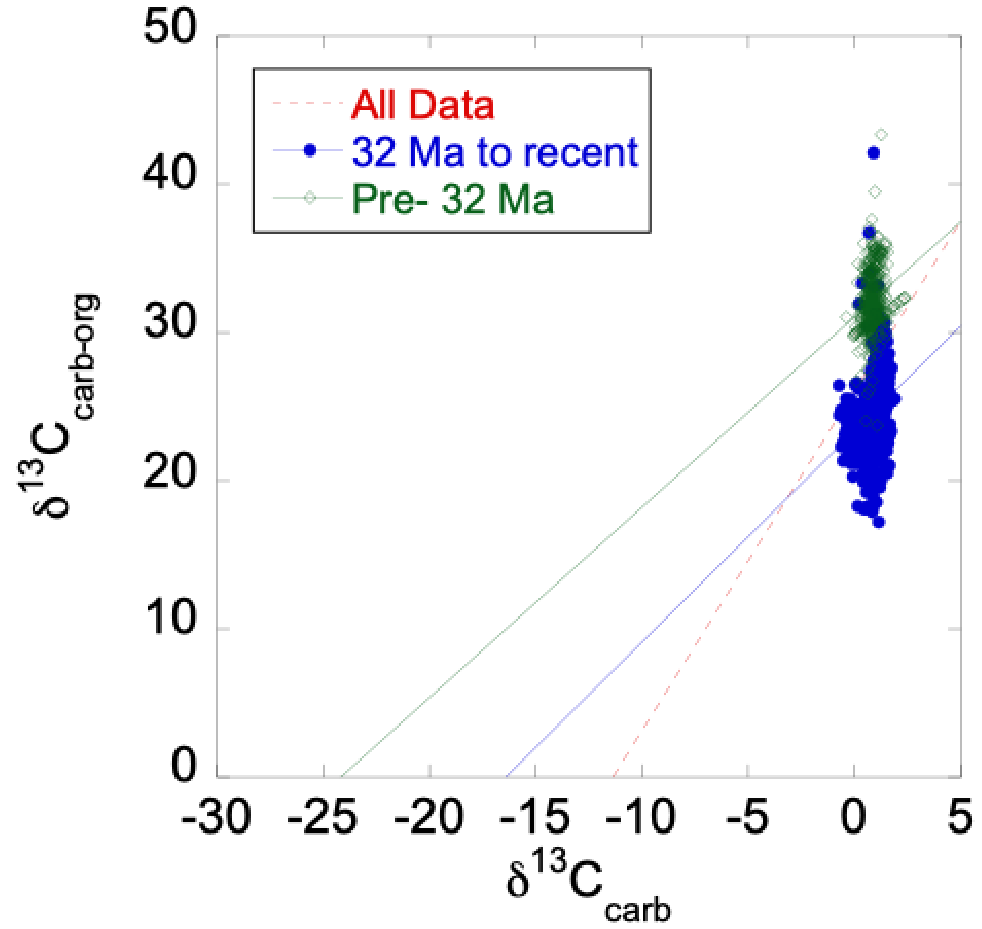

Fig. S1. A plot of $\delta^{13}C_{carb}$ v $\delta^{13}C_{carb-org}$ separating the data by pre (green) and post 32 Myrs (blue) compared to the whole dataset. The intercepts at $^{13}C_{carb-org}$ = 0 indicate the Rubisco fractionation factor for the different times and are suggestive that the Rubisco fractionation factor of the dominant alkenone producing phytoplankton diminished from ~ 24 ‰ to ~ 16 ‰ across the Eocene-Oligocene Boundary. That this shift is approximately ~ 10 ‰ could also imply that, coincidentally, the younger alkenone producing algae were better able to utilize the 10 ‰ heavier HCO_3^- ion for photosynthesis compared to CO_2.

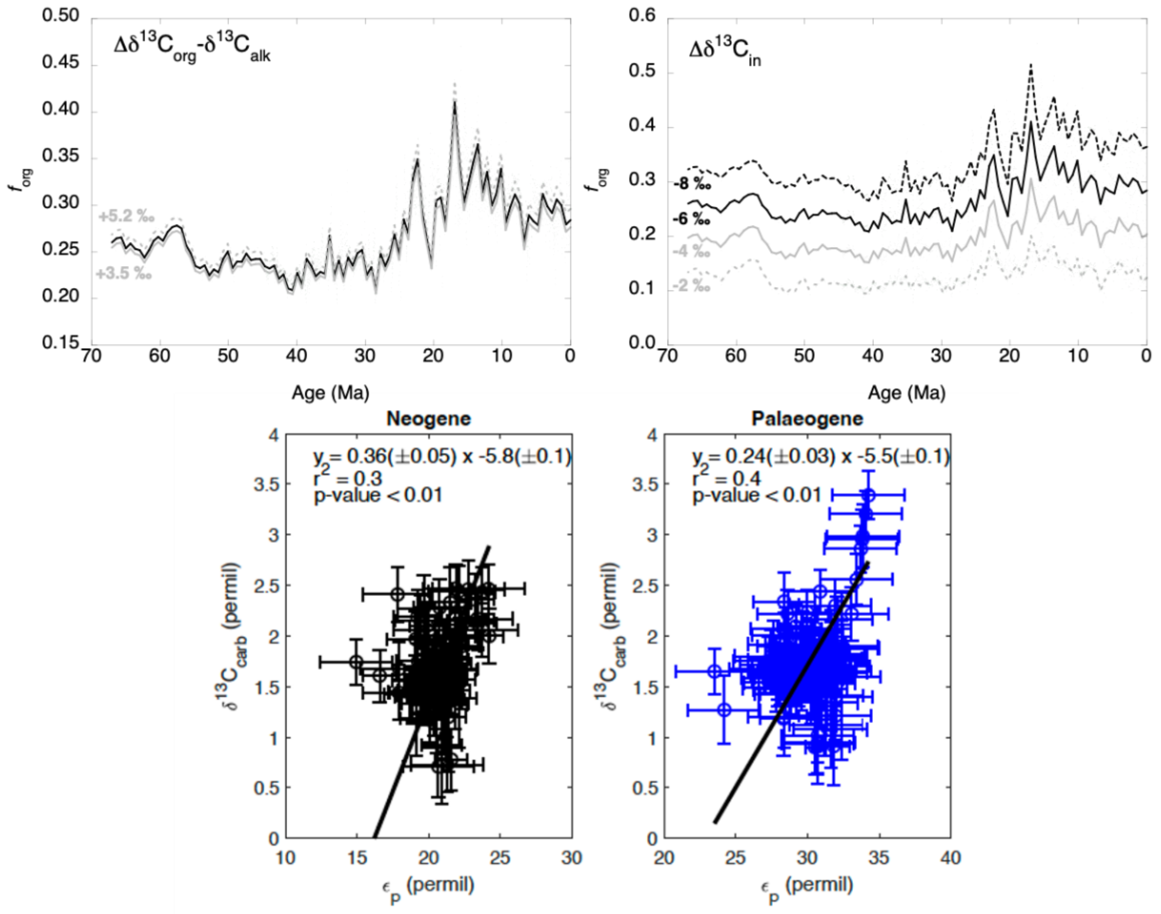

Fig. S2 Exploring the sensitivity of the f_{org} calculation to variation in the $\delta^{13}C_{in}$ and to the offset between the alkenone and biomass $\Delta\delta^{13}C_{org}$- $\delta^{13}C_{alk}$ (a, b) and using a regression analysis to constrain the $\delta^{13}C_{in}$ (c, d). a) Derivation of f_{org} with assumptions for $\delta^{13}C_{in}$ ranging from -8 ‰ (dashed black line), -6 ‰ (solid black line), -4 ‰ (solid grey line) and -2 ‰ (dashed grey line), and with $\Delta\delta^{13}C_{alk}$- $\delta^{13}C_{org}$ ranging from +3.5 ‰ (solid grey line), to +4.2 ‰ (solid black line) to +5.2 ‰ (dashed grey line). (c, d) The data has been binned into 300 kyr time bins (3x present residence time ~100 kyr), before performing a "York" regression (23) taking into account the standard deviation of each point in both x and y direction).. Despite the low r^2, the regressions are suggestive of a slope (= f_{org}) that is greater in the Neogene than the Paleogene. The y-intercepts at -5.8 and -5.5 ‰ are greater than the range of (4) (-8±1.9 ‰), but are in line with a -6 ‰ of (20).

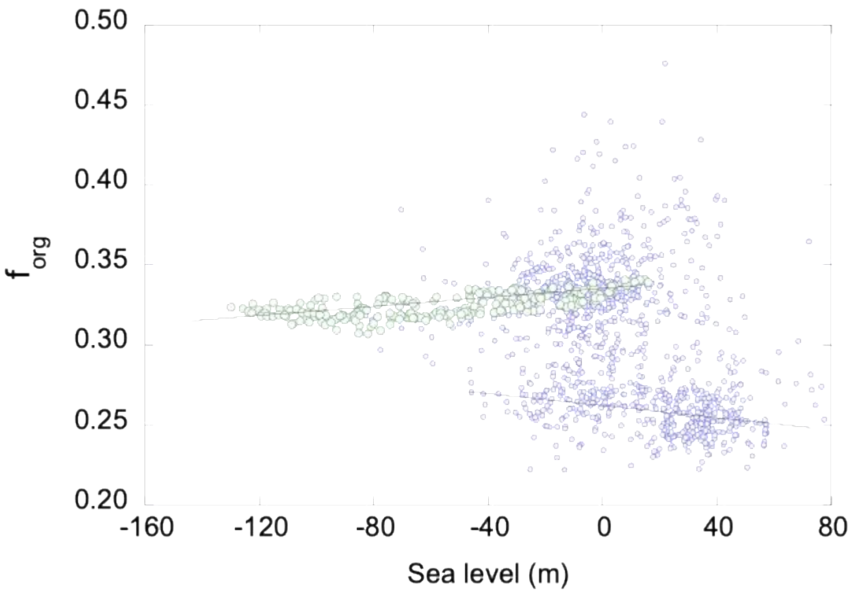

Fig. S3 A scatter plot of f_{org} versus sea level to confirm the declining trend for f_{org} with declining sea level for the Cenozoic dataset (open blue circles), and for the Pleistocene calculated using the alkenone $\delta^{13}C$ compilation together with the $\delta^{13}C$ of deep Pacific benthic foraminifera (24) and sea level for the last 430 kyrs (25)

SI References

1. A. Ridgwell, R. Zeebe, The role of the global carbonate cycle in the regulation and evolution of the Earth system, *Earth Planet. Sci. Letts* **234**,299-315 (2005).
2. G. Li and H. Elderfield, Evolution of carbon cycle over the last 100 million years. *Geochim. Cosmochim. Acta,* **103**, 11-25 (2013).
3. J. K. Caves, A. B. Jost, K. V. Lau, K. Maher, Cenozoic carbon cycle imbalances and a variable weathering feedback. *Earth Planet. Sci. Lett.* **450**, 152–163 (2016). https://doi.org/10.1016/j.epsl.2016.06.035.
4. L. A. Derry, Closing the Geologic Carbon Cycle. *Proc. Nat. Acad. Sci.* **121**, e2409333121 (2024).
5. A. Dutkiewicz, R. D. Müller, The history of Cenozoic carbonate flux in the Atlantic Ocean constrained by multiple regional carbonate compensation depth reconstructions. Geochem. Gephys. Geosys. 23, (2002). https://doi.org/10.1029/2022GC010667
6. B. P. Boudreau, Y. Luo, Retrodiction of secular variations in deep-sea CaCO3 burial during the Cenozoic. *Earth Planet. Sci. Lett.* **474**, 1–12 (2017). https://doi.org/10.1016/j.epsl.2017.06.005.
7. B. N. Opdyke, B. H. Wilkinson, Surface area control of shallow cratonic to deep marine carbonate accumulation. *Paleoceanogr*. **3**, 685–703 (1988). https://doi.org/10.1029/PA003i006p00685.
8. L. R. Kump, M. A. Arthur, Global chemical erosion during the Cenozoic: Weatherability balances the budget in Tectonic Uplift and Climate Change, W. Ruddiman, Ed. (Plenum, New York, 1997), pp. 399–426, https://doi.org/10.1007/978-1-4615-5935-1 (1997).
9. T. Salles, L. Husson, T. Trung Nguyen, A. Vila-Concejo, J. Leonard, A.P. Da Silva, J.M. Webster, & F. Giraud, Carbonate burial regimes, the Meso-Cenozoic climate, and nannoplankton expansion, Proc. Natl. Acad. Sci. U.S.A. 122 (49) e2516468122, (2025). https://doi.org/10.1073/pnas.2516468122

10. J. M. Husson, S. E. Peters, Shifting carbonate burial between oceanic and continental crust across Earth history. *Earth Planet. Sci. Lett.* **677**, 119810 (2026). https://doi.org/10.1016/j.epsl.2025.119810.
11. E. A. Laws, B. N. Popp, R. R. Bidigare, M. C. Kennicutt, S. A. Macko, Dependence of phytoplankton carbon isotopic composition on growth rate and [CO2]aq: Theoretical considerations and experimental results, Geochim. Cosmochim. Acta, 59, 1131-1138, (1995)
12. B. N. Popp, E. A. Laws, R. R. Bidigare, J. E. Dore, K. L. Hanson, S. G. Wakeham, Effect of Phytoplankton Cell Geometry on Carbon Isotopic Fractionation, Geochim. Cosmochim. Acta, 62, 69-77, (1998)
13. Hedges, J. I., and R. G. Keil (1995), Sedimentary organic matter preservation: an assessment and speculative synthesis, *Mar. Chem.*, 49, 81–115.
14. J. Jasper, J. M. Hayes, A carbon isotope record of CO2 levels during the late Quaternary. Nature 347, 462–464 (1990). https://doi.org/10.1038/347462a0
15. B. N. Popp, F. Kenig, S. G. Wakeham, E. A. Laws, R. R. Bidigare Does growth rate affect ketone unsaturation and intracellular carbon isotopic variability in Emiliania huxleyi?, Paleoceanography, 13 , 35–41, (1998) doi:10.1029/97PA02594.
16. M. Pagani, The alkenone–CO2 proxy and ancient atmospheric carbon dioxide, Phil. Trans. R. Soc. Lond. A 360, 609–63210.1098/rsta.2001.0959 (2002)
17. The Cenozoic CO_2 Proxy Integration Project (CenCO2PIP) Consortium* Toward a Cenozoic history of atmospheric CO_2. *Science*, **382**, eadi5177(2023) doi:10.1126/science.adi5177
18. E. B. Wilkes, R. B.Y. Lee, H. L.O. McClelland, R.E.M. Rickaby, A. Pearson, Carbon isotope ratios of coccolith–associated polysaccharides of Emiliania huxleyi as a function of growth rate and CO2 concentration, Organic Geochemistry, Volume 119, 2018, Pages 1-10, (2018)
19. J. M. Hayes, Fractionation of Carbon and Hydrogen Isotopes in Biosynthetic Processes* Reviews in Mineralogy and Geochemistry 43, 225–277, (2001) https://doi.org/10.2138/gsrmg.43.1.225
20. J. M. Hayes, H. Strauss, A. J. Kaufman, The abundance of ^{13}C in marine organic matter and isotopic fractionation in the global biogeochemical cycle of carbon during the past 800 Ma. Chem. Geol. **161**, 103-125 (1999).
21. D. H. Rothman, J. M. Hayes, R. E. Summons, Dynamics of the Neoproterozoic carbon cycle. *Proc. Nat. Acad. Sci.* **100**, 8124-8129 (2004).
22. T. Westerhold et al., An astronomically dated record of Earth's climate and its predictability over the last 66 million years. *Science* **369**, 1383–1387 (2020).
23. D. York, N. M. Evensen, M. L. Martínez, J. De Basabe Delgado, Unified equations for the slope, intercept, and standard errors of the best straight line. *American Journal of Physics* **72**, 367-375 (2004).
24. L.E. Lisiecki, M.E. Raymo, W.B. Curry, Atlantic overturning responses to Late Pleistocene climate forcings. *Nature*, **456**, 85-88, doi:10.1038/nature07425 (2008)
25. R. M. Spratt, L. E. Lisiecki, A Late Pleistocene sea level stack, version 2. PANGAEA, https://doi.org/10.1594/PANGAEA.979830 (2025):
